# Binding of permanganate anion to pentaammineazidocobalt(III) cation in solution and solid phases: synthesis, characterization, X-ray structure, and genotoxic effects of [Co(NH_3_)_5_N_3_](MnO_4_)_2_⋅H_2_O

**DOI:** 10.3906/kim-2012-66

**Published:** 2021-08-27

**Authors:** Ritu BALA, Jinkwon KIM, Vinit PRAKASH

**Affiliations:** 1 Department of Chemistry, Guru Nanak Dev University, Amritsar, Punjab India; 2 Department of Chemistry, Kongju National University, Shinkwan, Kongju, Chungman South Korea; 3 Department of Chemistry, Maharishi Markandeshwar University, Sadopur, Ambala, Haryana India

**Keywords:** Anion binder, pentaammineazidocobalt(III), coordination chemistry, genotoxicity, IR spectroscopy, X-ray crystallography

## Abstract

A pentaammineazidocobalt(III) complex, [Co(NH_3_)_5_N_3_](MnO_4_)_2_XH_2_O has been synthesized by an one-pot synthesis method. It was characterized by studies such as infrared (IR) and UV-visible spectroscopy. The single crystal X-ray structure analysis revealed that the title complex crystallizes in space group Cc. The cobalt center is six coordinated with slightly octahedral geometry. The supramolecular architecture is also formed by intermolecular N-H…O (anion and H_2_O) and Mn-O…O-H hydrogen bonds. The binding property of the cation, [Co(NH_3_)_5_N_3_]^2+^ with the anion, MnO_4_^–^ has also been determined (in solution phase) with the help of UV-visible spectroscopic titrations. Further, the genotoxic effects of KMnO_4_, [Co(NH_3_)_5_N_3_]Cl_2_ and [Co(NH_3_)_5_N_3_](MnO_4_)_2_XH_2_O were studied using *Allium cepa* root chromosomal aberration assay and it revealed that the genotoxicity of the newly synthesized complex is 1.97–1.76 fold, which is less compared to KMnO_4_. The order of genotoxic potential has been observed to be KMnO_4 _> [Co(NH_3_)_5_N_3_](MnO_4_)_2_XH_2_O > [Co(NH_3_)_5_N_3_]Cl_2_.

## 1. Introduction

Potassium permanganate is an exceptionally strong oxidizing agent, violet colored, crystalline, odorless substance and easily available from any pharmaceutical shop. It is primarily used as disinfectant [1,2], deodorizing [34], astringent [5,6] as well as to remove iron, manganese and hydrogen sulfide from water [7]. Along with its commercial importance KMnOu - _4 _is also known for its detrimental effects like, if it is orally injected, it may lead to the death of the patient due to tissue contraction, necrosis and hepatorenal toxicity [8,9]. The reason behind this is the oxidative injury from free radicals generated by the absorbed permanganate ion [1012]. Genotoxicity of KMnO -_4 _solution has been shown using a micro technique of the Ames test [13,14]. It was also reported to cause the DNA damage in human peripheral blood lymphocytes (with a dose dependent response in single-cell gel assay (SCGA)) [15,16]. Therefore, it is necessary to eliminate or reduce the toxic effect of KMnO _4_ from the environment. In order to prevent its deleterious impacts on ecosystems and public health, several extraction and recovery methods for KMnO_4_ have been already reported like electrochemical sensors [7], chemical reduction [17] and solvent extraction [18,19]. The recovery methods are rather costly and time consuming. Consequently, to remove or reduce the toxic effects of KMnO _4, _there is need of a method that is simple, effective, inexpensive and environmental friendly. Keeping this in mind, pentaammineazidocobalt(III) cation has been explored as an anion binder for permanganate ion in aqueous medium (green chemistry). 

Moreover, if we look back the literature there are sporadic reports regarding synthesis and crystal structure determination of [Co(NH_3_)_5_N_3_]^2+^ complex ions. The first azido complex of this cation was came into the limelight in 1934 [20] and on, Linhard group prepared a series of complexes containing [Co(NHlatter_3_)_5_N_3_]^2+^ [21,22]. Further, there are only four reports present in literature on X-ray structure determination of complexes containing [Co(NH _3_)_5_N_3_]^2+^ by three groups Palenik, [23] Blaurock [24] and Bala [25,26]. 

Therefore, it was thought to be of interest to synthesize and study the [Co(NH a_3_)_5_N_3_]^2+^ complex ion containing oxoanion, permanganate. This paper reports the synthesis, characterization,single crystal X-raystructure determination of [Co(NH _3_)_5_N_3_](MnO_4_)_2_.H_2_O. The binding properties of pentaammineazidocobalt(III) cation with permanganate anion have been determined by UV-visible titrations. The comparative study of genotoxic effects of KMnO _4_, [Co(NH_3_)_5_N_3_]Cl_2 _and [Co(NH_3_)_5_N_3_](MnO_4_)_2_.H_2_O have been determined using genotoxicity assay in Allium cepa root tip cells, which also has shown the importance to bind MnO_4_ in aqueous medium. 

## 2. Materials and methods

The reagents (AR grade of Merck) were used as such without any additional purification. [Co(NH_3_)_5_N_3_]Cl_2 _was prepared according to the method reported by Linhard et al. [27]. Cobalt was determined by standard method [28] and H, N was estimated microanalytically by automatic Eager 300 elemental analyzer. Solubility of the newly synthesized complex was measured at temperature 25 ± 2 ºC. The Shimadzu 1800 spectrophotometer was used for UV-visible spectra using quartz cuvette (water as solvent). For spectra, Varian Resolution Pro 660 FT/IR spectrophotometerwas instrument,Infrared utilized as KBr pellets. XRD was recorded on a x-pert-pro PANalytical (Cu-Kα radiation, λ = 0.15418 nm) having angle range 560was t- ^o^. The thermal was recorded in temperature range from 25 to 500behaviour(with ramp of 10 ^o^C rate) using TGA/DTA/NETZSCH STA 449F1 instrument, with ramp of 10 ^o^C . 

### 2.1. Synthesis of [Co(NH_3_)_5_N_3_](MnO_4_)_2_×H_2_O

Hot (6070 -^o^C temperature) aqueous solution (30 mL) of pentaammineazidocobalt(III) chloride (0.50 g, 0.0019 mol) was reacted with a hot (6070 -^o^C temperature) aqueous solution (15mL) of potassium permanganate (0.615g, 0.0077 mol) at room temperature. The mixture of solutions was allowed to cool slowly by keeping it overnight which resulted in formation of crystals. The crystals were filteredair dried at room temperature. The violet clear supernatant solution (after day) gave second crop of crystals. The overall yield was 0.774 g (90%). The melting point of the newly synthesized complex was observed to be 415 K (dec.). Anal. Calcd. for newly synthesized complex, [Co(NH , colouredone _3_)_5_N_3_](MnO_4_)_2_ **×** H_2_O: Molecular weight 442.03 g/mol: Co, 13.33; N, 25.35; H, 3.88%. Found: Co, 13.32; N, 25.31; H, 3.85 %. IR (cm^1^^-^), ν 3460 (OH); ν _a _3230 (NH_3_); ν_s _3194.23 (NH_3_); ν 2036.90 (N_3_); δ_d _1699.34 (NH_3_); δ_s _1307.78 (NH_3_); ρ_r _833.28 (NH_3_); ν 904.64 (MnO_4_). UV-vis (solution): λ_max_, 303, 504, 523, 543 and 562 nm.

### 2.2. General procedure for Job’s plot by UVvis method

The stock solutions (1×10^5 ^^-^M) of receptor, [Co(NH_3_)_5_N_3_]Cl_2_ and the guest, KMnO_4_ were prepared in aqueous medium. The absorbance in each case with different receptor (10:0)-guest (0:10) ratio but equal in volume (10 mL) was recorded. The Job’s plots were drawn to find out the stoichiometries using continuous variation method at ) *λ*_max_ 313 nm for manganate.

### 2.3. UV-visible titrations

The binding tendency of complex cation for manganate anion was determined using UVvisible titrations. The stock solutions of [Co(NH–_3_)_5_N_3_]Cl_2_ and KMnO_4_ were prepared in concentration 1X10^4^^-^ M and 1 X 10^2^^-^ M respectively in aqueous medium. The titration were performed by adding increments of 5 μL solution of anion (each addition was made after 1 ) into stock solution (2 mL) of [Co(NHminute_3_)_5_N_3_]Cl_2_ in a quartz cuvette (optical path length 1 cm). All absorption spectra were recorded, saved and used to find the binding constant by fitting the data with the global analysis program Hypspec20141 www.hyperquadcouk/HypSpechtml [29]

### 2.4. Crystal structure determination

Single-crystal diffraction data for [Co(NH_3_)_5_N_3_](MnO_4_)_2_ **×** H_2_O have been collected on a Bruker AXS SMART diffractometer equipped with CCD detector. More than a hemisphere of data was collected on each crystal over three batches of exposure using MoK *_a_* radiation (l = 0.71073 Å). A fourth set of data was measured and compared to the initial set to monitor and correct for decay, which was negligible. Data processing was performed using the program SAINT [3].The absorption correction was done using an empirical method (SADABS) [3]. The structure was solved by the direct method and refined by the full-matrix least-squares method on all F12 ^2^ data using SHELX-97 [3]. All other information regarding the refinement is also recorded in Table 1. 

**Table 1:** Crystal structure refinement data of [Co(NH3)5N3](MnO4)2×H2O.

Empirical formula	H17CoMn2N8O9 (Figure 5)
Formula weight	442.03
Temperature (K)	293(2)
Crystal system, space group	Monoclinic, Cc
Unit cell dimensions	a = 9.4996(15) Ǻ
	b = 12.110(2) Ǻ
	c = 12.704(2) Ǻ
	β = 107.733(9)º
Volume (Ǻ3)	1392.0(4)
Z, calculated density (g/cm3)	4, 2.109
Absorption coefficient (mm–1)	3.020
F(000)	888
θ range for data collection (◦)	2.81–38.83
Index ranges	–16 ≤ h ≤ 16, –17 ≤ k ≤ 21, –22 ≤ l ≤ 16
Reflections collected	16219
Independent reflections	6188 [Rint = 0.0463]
Data/restraints/parameters	6188/2/181
Goodness-of-fit on F2, (S)	1.010
Final R indices, 288 reflections [I > 2σ ( I )]	R1 = 0.0439 , wR2 = 0.0826
R indices (all data)	R1= 0.0865, wR2 = 0.0967

### 2.5. Genotoxicity of the compounds

#### 2.5.1. Genotoxic potential of KMnO 4, [Co(NH3)5N3]Cl2 and [Co(NH3)5N3](MnO4)2×H2O

The enotoxic potential of the compounds viz., KMnOG_4_, [Co(NH_3_)_5_N_3_]Cl_2_ and [Co(NH_3_)_5_N_3_](MnO_4_)_2_ **×** H_2_O was estimated using *Allium cepa* (onion) root chromosomal aberration assay. To evaluate genotoxicity, fresh bulbs of *Allium cepa* were peeled off and the primary roots were plucked with the help of forcep without disturbing the primordia. The onion bulbs were placed on the Couplin jars containing distilled water for germination of fresh roots. After the growth of roots to 0.51 cm, they were treated with different concentrations viz., 50, 100, 250 and 500 ppm of solution of all the three compounds i.e. KMnO - _4_, [Co(NH_3_)_5_N_3_]Cl_2_ and [Co(NH_3_)_5_N_3_](MnO_4_)_2_ **×** H_2_O for 3h. The roots were then plucked and fixed in farmer’s fluid (3:1:: ethanol:glacial acetic acid). The slides were prepared using squash method and were screened under microscope to score different types of aberrations. Three slides were scored for each concentration with at least 5070 dividing cells. 

#### 2.5.2. Genotoxic potential of ead cetate

Estimation of genotoxic potential lead (0.5 ppm) was evaluated as per the protocol mentioned in section 2.5.1 

#### 2.5.3. Antigenotoxicity of [Co(NH3)5N3]Cl2, [Co(NH3)5N3](MnO4)2×H2O against lead

In order to see the sequential effects of newly synthesized compounds against lead induced genotoxicity, two modes of treatment i.e. pre and post were followed after germination of roots. In pretreatment, the onion roots were treated with [Co(NH_3_)_5_N_3_]Cl_2_ for 3h followed by treatment with lead while in post treatment, roots were first exposed to lead solution (0.5 ppm) and then to [Co(NH _3_)_5_N_3_]Cl_2_. After treatment, the slides were prepared using standard protocol and screened for chromosomal aberrations in *Allium* root tip cells.

## 3. Results

### 3.1. Synthesis

The single pot synthetic approach was used for this synthesis of title complex. Under this approach pentaammineazidocobalt(III) chloride and potassium permanganate were reacted in 1:2 molar ratio in hot aqueous medium with the expectation of [Co(NH_3_)_5_N_3_](MnO_4_)_2 _complex The violet complex, [Co(NHcoloured_3_)_5_N_3_](MnO_4_)_2_ was formed according to expectation in addition to one lattice water molecule. The chemical composition of new cobalt(III) complex was initially confirmed by elemental analyses which corresponds to chemical formula [Co(NH_3_)_5_N_3_](MnO_4_)_2_×H_2_O. The methodology used for the preparation of title complex is similar as reported earlier by various groups for the preparation of [Co(NH_3_)_5_N_3_]^2+^ containing complexes [2026]. 

-Solubility product

The newly synthesized complex is insoluble in organic solvent (CCl _4_, CHCl_3_) but soluble in inorganic solvent (DMSO, MeCN) indicating the ionic . Solubility of ionic complex in water differs to a great extent and on the basis of solubility criterion, the ionic complexes are classified into three categories (a) solubility > 0.1 M (soluble) (b) solubility between 0.01 and 0.1 M (slightly soluble) (c) solubility < 0.01 M (sparingly soluble). The solubility product, Kbehaviour_sp_ of new cobalt(III) complex is 5.41×10^5^^-^ at 25 ± 2 , indicating its slight solubility in water. The order of solubility was observed as: KMnOºC_4_> [Co(NH_3_)_5_N_3_]Cl_2_> [Co(NH_3_)_5_](MnO_4_)_2_×H_2_O. 

### 3.2. UVisible spectrum

**Figure 1:**
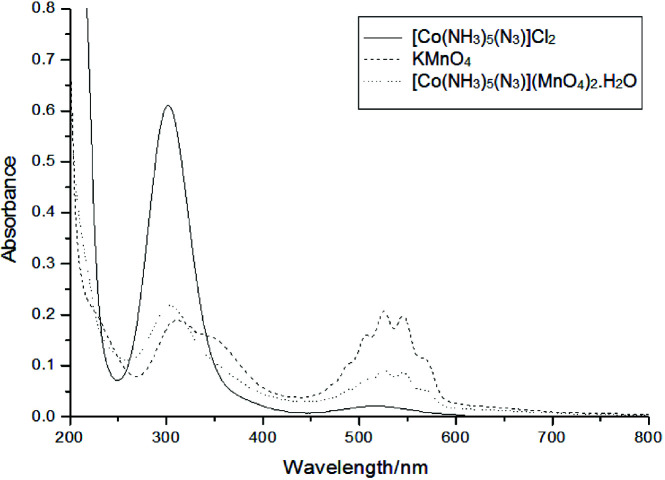
UV-visible spectra of [Co(NH_3_)_5_N_3_]Cl_2_, KMnO_4_, and [Co(NH_3_)_5_N_3_] (MnO_4_)_2_ H_2_O.

All the UVisible spectra were recorded in water. The two transitions approximately at 515 (/V ^1^A_1g_®T _1g_)and 302 nm (^1^A_1g_®^1^T_2g_) were described [2527] for pentaammineazidocobalt(III) cation containing complexes, producing the familiar dark violet . For [Co(NH-colour_3_)_5_N_3_]Cl_2_ these transitions were observed at 518 and 299 nm (See Figure 1). However for KMnO_4_, peaks were observed at 509, 527, 547, 569 and 313 nm along with one shoulder around 354 nm (see Figure 1) although it is dfive ^0^ system, Mn(+7). These peaks are observed due to ligand to metal charge transfer bands (LMCT) i.e an O lone-pair electron is promoted into a low-lying empty e orbital of metal. But for [Co(NH,_3_)_5_N_3_](MnO_4_)_2_×H_2_O, the maxima were observed at 303, 504, 523, 543 and 562 nm (ee Figure 1). The disappearance of peaks at 313 and 515 nm of MnOS _4_ and [Co(NH^-^_3_)_5_N_3_]^2+^ respectively may be due to overlapping of d-d transitions of Co(III) with charge transfer bands of MnO _4_^-^i.e., 313 nm of MnO _4_ (charge transfer band) merged with 299 nm of Co(III) cation (d-d transition) and 518 nm of Co(III) cation (d-d transition) merged with 527 nm MnO^-^_4_^-^(charge transfer band). 

### 3.3. Jobs lot and UVisible titrations

**Figure 2:**
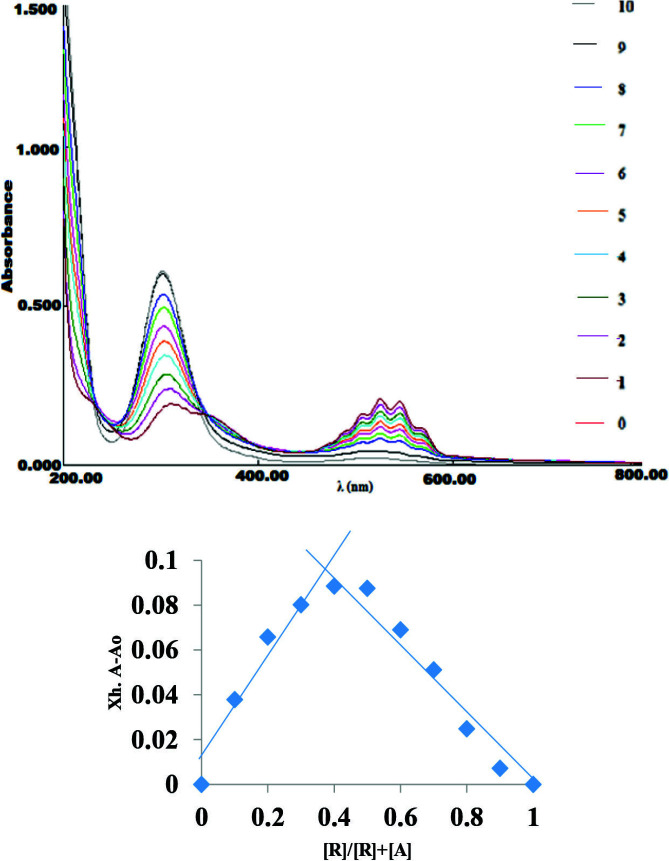
(a) UV/vis titration spectra of receptor, [Co(NH_3_)_5_]Cl_2_ recorded in water (1  10^-5^ M) after the addition of 0–1equivalents of KMnO4. (b) Job’s plot (using continuous variation method of receptor with KMnO_4_ at λ_max_ = 313 nm.

After taking the UV-visible spectra of complex cation and anion (Figure 2a), the stoichiometry was determined by Job’s plot (using continuous variation method) at different λ _max _313 nm. Jobs plot is plotted between ΔA* XR or XG (mole fraction of receptor or guest) (along y-axis) and XR or XG (along x-axis) in the mixtures where the total concentrations of receptor and guest remain constant. It is used to identify the stoichiometry of the complex. The maximum were observed at 0.35 mole fraction with respect to receptor respectively, which implied the stoichiometry to be 1:2 (see Figure 2b).

**Figure 3:**
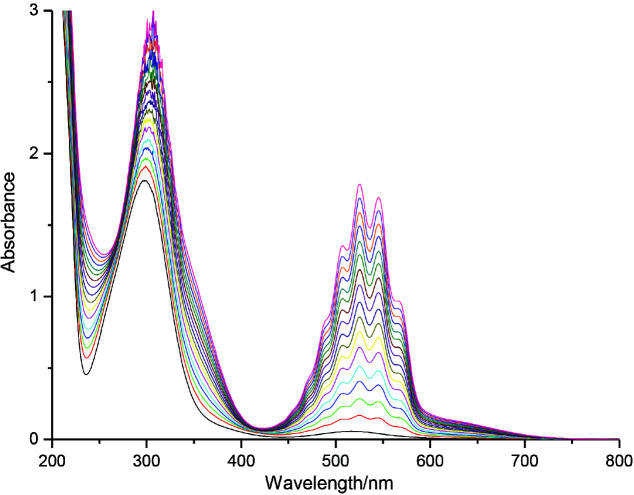
UV-vis titration spectra of receptor, [Co(NH_3_)_5_N_3_]Cl_2_ recorded in water (1  10^-4^ M) after the addition of KMnO4 solution (1   10^-2^ M).

 For investigating the binding properties of [Co(NH_3_)_5_N_3_]^2+^ for permanganate anion (MnO_4_^-^), titration experiments were performed according to the procedure given in the experimental section. After constant additions of KMnO_4_ in receptor (R) a gradual red shift (bathochromic shift) is observed from absorption maxima 299306 nm along with hyperchromic shift in all the maxima (which were observed at 507, 525, 545 and 564 nm, Figure 3). The disappearance of peaks at 313 and 515 nm of MnO to mabsorbationSee _4_ and [Co(NH^-^_3_)_5_N_3_]^2+^ respectively and 7 nm red shift in the presence of permanganate anion could result in the interaction between receptor and anion. Through the spectral fitting of titration data the log β value was observed at 8.8128 ± 0.02 with the formation stoichiometry 1:2 for MnO_4_^-^ This stoichiometry was also matched with the stoichiometry obtained from the Job’s plot titrations (Figure 2 b). 

### 3.4. Infrared spectrum

The infrared spectrum of [Co(NH_3_)_5_N_3_](MnO_4_)_2_×H_2_O has been recorded in the range 400 to 4000 cm^1^^-^ and interpretations have been made on the basis of earlier reported literature [25,26,3]. In new cobalt(III) complex, the stretching vibration frequencies were lower for the coordinated NH 4_3_ molecules than those of the free NH_3_. This lowering might be due to the formation of N-H…O type of hydrogen bonds which weaken the N-H bonds. It has been observed that the rocking vibrations (r_r_), symmetric deformation (d_s_), degenerate deformation (d_d_), and antisymmetric (n_as_) and symmetric (n_s_) stretch appeared in the regions of 800900, 13701200, 16501550, and 3400---3000 cm-^1^^-^ respectively [25] for [Co(NH_3_)_5_N_3_]Cl_2_ which was comparable with [Co(NH _3_)_5_N_3_](MnO_4_)_2_×H­_2_O. For ionic MnO_4_ a sharp band was observed at 904 cm^-^^1^^-^ for [Co(NH_3_)_5_N_3_](MnO_4_)_2_×H­_2_O which is comparable with the band observed in case of potassium permanganate i.e., 902 cm^1^^-^ [3]. 

### 3.5. TG/DT/DTG nalysis

**Figure 4:**
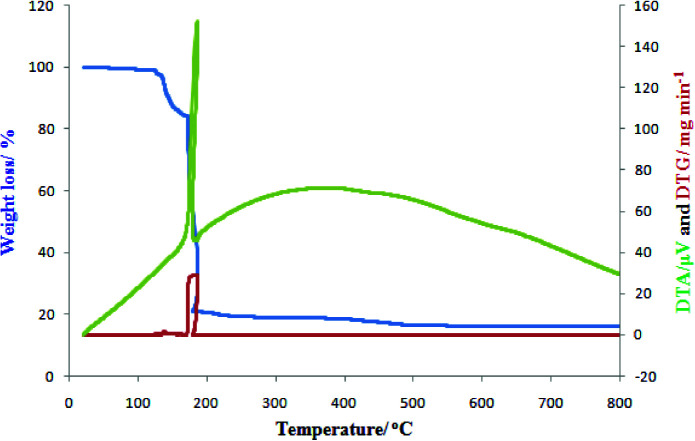
TG, DT, and DTG analyses plot of [Co(NH_3_)_5_N_3_](MnO_4_)_2_ H_2_O.

Thermal stability of title complex was examined between 20 and 800under nitrogen flow (Figure 4). The decomposition of complex started with the loss of water and azide in two steps, one at 117 and other at 125 ^o^C ^o^C. The complete loss (water and azide) occurs at 159^o^C ^o^(13.6 %) followed by loss of ammonia and decomposition of MnOC _4_^-^ up to temperature 183 . Both these decomposition steps were exothermic in nature (ee Figure 4). After that not much change in weight loss was observed.

### 3.6. Single crystal X-ray diffraction (SCXRD)

**Figure 5:**
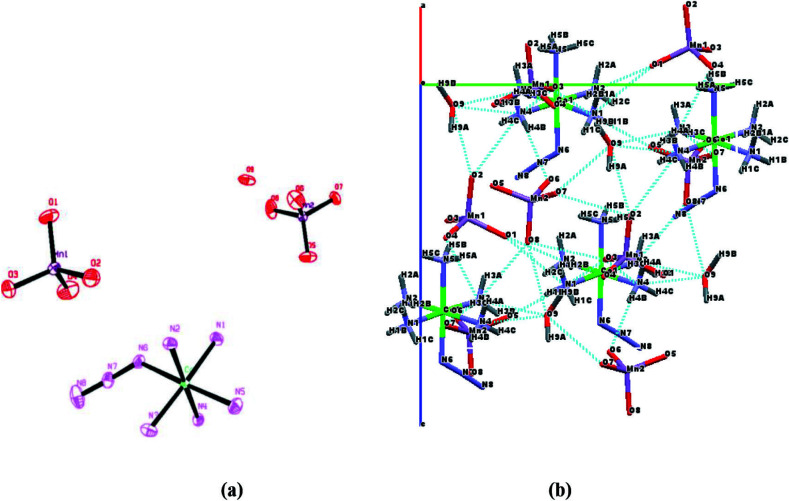
(a) ORTEP diagram of [Co(NH_3_)_5_N_3_](MnO_4_)_2_ H_2_O (30% ellipsoidal probability, H- atoms are omitted for clarity) (b) Crystal packing diagram (down to a*-axis) for [Co(NH_3_)_5_N_3_](MnO_4_)_2_.H_2_O showing hydrogen bonding interactions. Dotted lines represent hydrogen bonds.

The X-ray crystal structure of the [Co(NH_3_)_5_N_3_](MnO_4_)_2_×H_2_O was unambiguously determined by single crystal X-ray crystallography. The crystal structure conclusively established the existence of single complex of composition, [Co(NH_3_)_5_N_3_](MnO_4_)_2_×H_2_O and also ruled out the possibility of a double salt and mixture of salts. Furthermore, this study revealed for the first time that it is an ionic complex which contains discrete ions, [Co(NH_3_)_5_N_3_]^2+^ and two MnO_4_ in addition to one lattice water molecule in the solid state. The numbering scheme and ORTEP diagram of [Co(NH^-^_3_)_5_N_3_](MnO_4_)_2_×H_2_O is shown in Figure 5a.

In [Co(NH_3_)_5_N_3_]^2+^, the cobalt(III) metal ion is surrounded by six nitrogen atoms originating from five coordinating ammonia molecules and one from azide molecule resulting in a nearly octahedral geometry. The Co–NH_3_ bond distances are in the range 1.958(4) 1.981(5) Å while cis H- _3_N–Co–NH_3_ bond angles are in the range 89.16(18)92.1(2)° and trans H - _3_N–Co–NH_3_ bond angles are in the range 177.4(2)178.86(18)°. The Co–N - _3_ bond distance is 1.955(5) Å, while cis H_3_N–Co–N_3_ and trans H_3_N–Co–N_3_ bond angles are in the range 86.1(2)91.3(2)° and 177.4(2) - 178.86(18)° respectively. This study showed that octahedron is slightly distorted. The bonding parameters of [Co(NH- _3_)_5_N_3_](MnO_4_)_2_ **×** H_2_O are also in according with the former reported [Co(NH_3_)_5_N_3_]^2+^ complex ion [2326]. The selected bond angles and bond lengths are listed in Table 2. In the pentaammineazidocobalt(III) azide, the average Co–NH-_3_ bond distance is 1.968 Å and average bond angles cis and trans H_3_N–Co–NH_3_ are 90.7° and179.8° respectively. 

**Table 2:** Bonding parameters, lengths (Å), and angles (°).

Co1-N1	1.967(4)	Mn1-O1	1.616(4)
Co1-N2	1.966(4)	Mn1-O2	1.579(5)
Co1-N3	1.958(4)	Mn1-O3	1.606(4)
Co1-N4	1.965(4)	Mn1-O4	1.598(4)
Co1-N5	1.981(5)	Mn1-O5	1.591(5)
Co1-N6	1.955(5)	Mn1-O6	1.602(4)
N6-N7	1.187(5)	Mn1-O7	1.620(4)
N7-N8	1.159(5)	Mn1-O8	1.586(5)
N1-Co1-N5	92.1(2)	N7-N6-Co1	121.5(3)
N2-Co1-N1	89.80(10)	N8-N7-N6	177.0(5)
N2-Co1-N5	90.2(2)	O2-Mn1-O1	109.9(3)
N3-Co1-N1	177.4(2)	O2-Mn1-O3	109.6(3)
N3-Co1-N2	90.11(17)	O2-Mn1-O4	108.4(3)
N3-Co1-N4	90.92(11)	O3-Mn1-O1	109.5(3)
N3-Co1-N5	90.4(2)	O4-Mn1-O1	109.3(3)
N4-Co1-N1	89.16(18)	O4-Mn1-O3	110.2(3)
N4-Co1-N2	178.86(18)	O5-Mn2-O6	111.7(3)
N4-Co1-N5	90.3(2)	O5-Mn2-O7	109.9(3)
N6-Co1-N1	86.1(2)	O6-Mn2-O7	108.3(2)
N6-Co1-N2	89.56(19)	O8-Mn2-O5	108.3(3)
N6-Co1-N3	91.3(2)	O8-Mn2-O6	108.6(3)
N6-Co1-N4	89.92(19)	O8-Mn2-O6	108.6(3)

In the MnO_4_^2^, the Mn-O bond distances are in the range 1.579(5)1.620(4) Å. The O-Mn-O bond angles are in the range 108.3(2)111.7(3)°. In literature, average Mn-O bond distance is 1.605 Å and average O-Mn-O bond angle is 109.5° for permanganate anion [35]. 

In the crystal lattice of title complex,^-^ - - supramolecular architecture is also formed (ee Table 3). It is formed by intermolecular hydrogen bonding between: 1) cation and anion (N-H(NHS_3_)…O (MnO_4_)), 2) solvent molecule (water) and cation (H-O (water)…H-N (cation)) and 3) solvent molecule (water) and anion (H-O-H (water)…O-Mn(anion)). The hydrogen bonding parameters are given in Table 3. This supramolecular architecture stabilizes the crystal lattice along with electrostatic forces of attractions between cations and anions. All kinds of hydrogen bonding interactions are shown in Figure 5b. 

**Table 3:** Hydrogen bonding parameters (Ǻ, °).

D-H…A	D - H	H...A	D...A	<D - H... A	Symmetry operations
N1-H1A…O(8)	0.89	2.36	3.149(7)	148	i
N1-H1B…O(5)	0.89	2.15	3.030(8)	170	ii
N1-H1C…O(6)	0.89	2.40	3.251(7)	160	iii
N2-H2B…O(7)	0.89	2.23	3.095(6)	164	iii
N2-H2C…O(5)	0.89	2.30	3.168(7)	164	i
N3-H3A…O(4)	0.89	2.50	2.966(8)	113’	iv
N3-H3B…O(9)	0.89	2.16	2.931(6)	144	i
N3-H3C…O(7)	0.89	2.10	2.971(7)	168	iv
N4-H4A…O(3)	0.89	2.54	2.934(6)	107	iv
N4-H4A…N(8)	0.89	2.28	3.147(7)	163’	vii
N4-H4B…O(6)	0.89	2.19	3.039(7)	159	iii
N4-H4C…O(2)	0.89	2.33	3.002(7)	133	vi
N4-H4C…O(9)	0.89	2.24	2.984(6)	141’	v
N5-H5A…N(8)	0.89	2.54	3.402(7)	162	vii
N5-H5C…O(6)	0.89	2.48	3.257(7)	146	ii
O9-H9A…O(2)	0.98	2.11	3.021(8)	154’	v

i = x, –y, 1/2 + z; ii = –1/2 + x, –1/2 + y, z; iii= –1/2 + x,1/2–y,1/2 + z; iv = ½ + x, 1/2–y, ½ + z; v = x,1–y, ½ + z; vi = x, y, 1 + z, vii = –1/2 + x, 1/2–y, –1/2 + z.

### 3.7. Powder X-ray diffraction (PXRD)

The simulated PXRD pattern of title compound is given in Figure 6a. The simulated XRD pattern was compared with the SCXRD pattern obtained from the cif file (Figure 6b ()() of the compound and they are similar to one another. This indicates that SCXRD pattern obtained from cif file have same crystalline phase with respect to their bulk materials. 

### 3.8. Genotoxic potential

#### 3.8.1. Genotoxic potential of KMnO_4_, [Co(NH_3_)_5_N_3_]Cl_2_ and[Co(NH_3_)_5_N_3_](MnO_4_)_2_×H_2_O (Table 4) 

KMnO_4 _was analyzed for its genotoxic potential and it was observed that its treatment on *Allium cepa* root tip cells resulted in physiological aberrations at all the concentrations (12,25,50 25 and 250 ppm) and total percent aberrant cells ranged from 64.37 80.48%. Ramsdorif et al. 2009 [15] has also reported the genotoxic effects of KMnO1 to _4_ in acidic conditions with a dose-response relationship as determined by single cell gel assay (SCGA). The authors reported that the conversion of MnOl_4_ to Mn^-^^2+^ resulted in the DNA damage in human lymphocytes. However, in our study we observe that KMnO_4_ did not induced only clastogenic aberrations. [Co(NH_3_)_5_N_3_]Cl_2 _has induced both physiological and clastogenic aberrations at 50, 100, 250 and 500 ppm) and itobserved that the genotoxic potential of [Co(NH _3_)_5_N_3_]Cl_2 _was less as compared to KMnO_4_. 

**Table 4:** Genotoxic potential of potassium permanganate (KMnO_4_), azidopentaaminecobalt(III) chloride ([Co(NH_3_)_5_N_3_]Cl_2_) and azidopentaaminecobalt(III) permanganate [Co(NH_3_)_5_N_3_](MnO_4_)_2._H_2_O in Allium cepa root chromosomal aberrations assay.

Treatment	Concentration (ppm)	TC		Number of cells with													TNAC(PA+CA)	
				Physiological aberrations (PA)									Clastogenic aberrations(CA)					
				Da	St	Si		Vg	Aa	Am	Total PA	% PA	Bg	Bk	Total CA	% CA	No.	%
-ve control (distilled water)	0	3333		2	2	-		-	2	1	12	8.00	-	1	1	1.13	13	8.66

+ve control Pb (0.5 ppm)	0.5	3064	158	11	39	9	-	-	5	-	64	40.50	8	2	10	6.33	74	46.83

KMnO4	12	2350	160	1	22	47	-	-	26	7	103	64.37	-	-	-	-	103	64.37
	25	2239	154	2	20	48	-	-	23	20	113	73.37	-	-	-	-	113	73.37
	50	2205	156	2	26	47	-	-	36	14	125	80.12	-	-	-	-	125	80.12
	125	2634	161	5	22	48	-	-	30	18	123	76.39	-	-	-	-	123	76.39
	250	2275	164	6	38	58	-	-	22	08	132	80.48	-	-	-	-	132	80.48

[Co(NH3)5N3]Cl2	50	2218	156	5	3	6	-	-	2	7	23	14.74	-	-	-	-	23	14.74
	100	1556	154	-	1	5	1	-	5	6	18	11.68	-	-	-	-	18	11.68
	250	3310	168	8	27	2	-	2	1	5	45	26.78	3	-	3	1.78	48	28.57
	500	2490	150	2	27	5	-	1	-	6	41	27.33	1	-	1	0.66	42	28.00
	
[Co(NH3)5N3](MnO4)2.H2O	50	1854	155	1	9	28	-	-	17	7	62	40.00	1	-	1	0.6	63	40.64
	100	2387	156	1	20	27	-	-	17	9	75	48.07	-	-	-	-	75	48.07
	250	2527	158	1	15	20	-	-	19	12	67	42.40	5	-	5	3.16	72	45.57
	500	2581	155	1	25	23	-	-	32	10	91	58.70	-	-	-	-	91	58.70

TC - total cells, TNAC - total number of aberrant cells, -ve control-negative control, +ve control-positive control, Da - delayed anaphase, Bk - chromosomal break, St - sticky, Si - spindle inhibition, Aa - abnormal anaphase, Am - abnormal metaphase, Lg - laggard/s, Vg - vagrant/s, Bg - chromatin bridge.

However it was interesting to note that when the newly synthesized complex was evaluated [Co(NH_3_)_5_N_3_](MnO_4_)_2_×H_2_O for its genotoxic potential at all concentration (at 25, 50, 100, 250 and 500 ppm), it induced almost half the number of chromosomal aberrations when compared to that of treatment with KMnO _4_. The probable reason behind this can be the less toxic behavior of newly synthesized complex, [Co(NH _3_)_5_N_3_](MnO_4_)_2_×H_2_O due to the fact of nonavailability of Mn ^2+^ ions in the solution. 

#### 3.8.2. Genotoxic potential of ead cetate

It was observed that lead acetate (0.5 ppm) has induced 46.83% total aberrant cells comprising of 40.50% physiological and 6.33 % clastogenic aberrations in *Allium cepa* root tip cells. The physiological aberrations included delayed anaphase/s (Da), stickiness (St), spindle inhibition (Sp), laggard/s (Lg) vagrant/s (Vg), abnormal anaphase/s (Aa) and abnormal metaphase/s (Am), while chromatin /s (Bg) and chromosomal break/s (Bk) constituted the clastogenic aberrations Figure 7. The genotoxicity of lead using various bioassays has been well documented [3Bridge[]63]. To evaluate if the complexes, [Co(NH-9_3_)_5_N_3_]Cl_2 _and [Co(NH_3_)_5_N_3_](MnO_4_)_2_×H_2_O could reduce the genotoxic potential of other carcinogenic chemicals (Table 5)

**Table 5:** Genotoxic potential of azidopentaaminecobalt(III) chloride ([Co(NH_3_)_5_N_3_]Cl_2_) and azidopentaaminecobalt(III) permanganate monohydrate ([Co(NH_3_)_5_N_3_](MnO_4_)_2.._H_2_O) against lead induced genotoxicity in Allium cepa root chromosomal aberrations assay.

Treatment		Concentration (ppm)	TC	TNDC	Number of cells with													TNAC(PA+CA)	
					Physiological aberrations(PA)									Clastogenic aberrations (CA)					
					Da	St	Si	Lg	Vg	Aa	Am	Total PA	% PA	Bg	Bk	Total CA	% CA	No.	%
[Co(NH3)5N3]Cl2	(Pre-treatment)	50	1551	163	2	2	62	-	-	6	4	76	46.62	1	-	1	0.6	77	35.03
		100	2652	158	-	07	12	-	-	3	10	32	20.25	-	-	-	-	32	20.25
		250	2842	156	-	23	14	-	-	6	07	50	32.05	-	-	-	-	50	22.00
		500	2093	156	-	13	08	-	-	5	5	31	19.87	2	-	2	0.13	33	21.15

	(Post-treatment)	50	2431	164	-	11	13	1	-	5	1	31	18.90	2	-	2	1.21	33	20.12
		100	3022	156	2	08	26	-	-	2	0	39	25.00	-	-	-	-	39	25.00
		250	1650	156	1	14	12	-	-	6	2	35	22.43	3	-	3	1.92	38	24.35
		500	2386	159	5	14	33	-	-	5	2	59	36.87	4	-	4	2.50	63	39.62
																	
[Co(NH3)5N3](MnO4)2.H2O	(Pre-treatment)	50	3541	156	1	5	75	-	-	1	4	86	55.13	-	-	-	-	86	55.13
		100	2025	155	4	19	57	-	-	4	8	92	59.35	-	-	-	-	92	59.35
		250	1235	155	1	12	47	-	-	-	8	68	43.87	-	-	-	-	68	43.87
		500	1053	153	4	19	52	-	-	4	10	89	58.17	-	-	-	-	89	58.17

	(Post-treatment)	50	1633	151	2	13	48	-	-	12	14	89	58.94	2	-	2	1.32	91	60.29
		100	1827	156	4	9	38	-	-	6	5	62	39.74	-	-	-	-	62	39.74
		250	2551	159	5	15	61	-	-	2	3	86	54.08	4	-	4	2.50	90	56.60
		500	1932	151	3	27	25	-	-	3	13	71	47.01	7	-	7	4.60	78	51.65

TC - total cells, TNAC - total number of aberrant cells, -ve control-negative control, +ve control-positive control, Da - delayed anaphase, Bk- chromosomal break, St - sticky, Si - spindle inhibition, Aa - abnormal anaphase, Am - abnormal metaphase, Lg - laggard/s, Vg-vagrant/s, Bg - chromatin bridge

The study was further carried out to explore the genotoxic potential of [Co(NH _3_)_5_N_3_]Cl_2 _and [Co(NH_3_)_5_N_3_](MnO_4_)_2_×H_2_O combined with lead acetate using pre and post-treatments. Although the newly synthesized complex [Co(NH _3_)_5_N_3_](MnO_4_)_2_×H_2_O showed less genotoxic potential as compared to KMnO_4_ but it did not reduce the effects of lead acetate when treated in combination with lead acetate during pre and post-treatments. On the other hand, the complex [Co(NH _3_)_5_N_3_]Cl_2 _reduced the toxicity of lead acetate when treated in combination with lead acetate during pre and post treatments. Thus, it can be concluded that the complex [Co(NH_3_)_5_N_3_]Cl_2_ can be explored as a potential anion receptor for the extraction of environmentally hazardous chemicals like lead.

## 4. Conclusion 

Lastly, it is concluded that crystals of [Co(NH_3_)_5_N_3_](MnO_4_)_2_ **×** H_2_O can be formed by reacting KMnO _4 _with [Co(NH_3_)_5_N_3_]Cl_2_ in aqueous medium. The X-ray crystallographic study of [Co(NH_3_)_5_N_3_](MnO_4_)_2_ **×** H_2_O shows the presence of discrete ions: one cation, two anion along with one water of crystallization in solid state. The binding ability of cation, [Co(NH_3_)_5_N_3_]^2+^ towards anion shows that ions remain intact in the aqueous medium also. Further, the genotoxic effects of potassium KMnO_4_, [Co(NH_3_)_5_N_3_]Cl_2 _and [Co(NH_3_)_5_N_3_](MnO_4_)_2_ **×** H_2_O have been determined using *Allium cepa* root chromosomal aberration assay. The relative genotoxicity of these compounds has following decreasing order: KMnO _4 _> [Co(NH_3_)_5_N_3_](MnO_4_)_2_ **×** H_2_O > [Co(NH_3_)_5_N_3_]Cl_2_. This order shows the significance to bind the MnO_4_ with [Co(NH^-^_3_)_5_N_3_]^2+ ^under the conditions of environmental prevalence of it.

## Supplementary material

Full lists of crystallographic data are available from the authors upon request. Crystallographic data for the structural analysis of the title compound has also been deposited at the FIZ, 76344 Eggenstein-Leopoldshafen (Germany), having CSD number 426476 (tel.: (49) 7247-808-205; fax: (49) 7247-808-666; E-mail: crysdata@fiz-karlsruhe.de).
